# Associations between the use of β-adrenoceptor acting drugs and the risk of dementia in older population

**DOI:** 10.3389/fneur.2022.999666

**Published:** 2022-12-22

**Authors:** Shishuang Cui, Fang Fang, Peijing Cui, Qianwen Jiang, Shaoqing Xu, Zhihong Xu, Jia'An Hu, Feika Li

**Affiliations:** ^1^Department of Geriatrics, Ruijin Hospital Affiliated to Shanghai Jiao Tong University School of Medicine, Shanghai, China; ^2^Medical Center on Aging, Ruijin Hospital Affiliated to Shanghai Jiao Tong University School of Medicine, Shanghai, China

**Keywords:** β-adrenoceptor, β2-agonists, β-antagonists, dementia, noradrenergic

## Abstract

**Objective:**

Age-related decline within the noradrenergic system is associated with reduced cognition. The β-adrenoceptors are widely expressed in the brain as well as in the peripheral. Medications targeting β-adrenoceptor activity have been widely used in older adults. The aim of this study was to explore the associations between β-adrenoceptor acting drugs and the risk of dementia in the older population.

**Methods:**

The subjects' information was collected from the electronic medical record (EMR) database. A propensity score matching strategy was conducted to select control participants for users of β2-agonists or β-antagonists. Logistic regression analysis was performed to estimate the risk of dementia with the use of β2-agonists or β-antagonists.

**Results:**

A total of 1,429 participants in the EMR database were included in the study. The use of β2-agonists was strongly associated with a decreased risk of dementia [OR = 0.324, 95% confidence interval (CI): 0.149–0.707, *P* = 0.005]. This decreased risk showed a statistically significant inverse time-dependent pattern (*P*_trend_ = 0.014). However, the use of non-selective β-antagonists significantly correlated with an increased dementia risk (OR = 1.961, 95% CI: 1.144–3.359, *P* = 0.014), although no time-dependent manner was found (*P*_trend_ = 0.220). There was no association between selective β1-antagonists usage and dementia risk (OR = 1.114, *P* = 0.625).

**Conclusion:**

The use of β-adrenoceptor acting drugs seems to be associated with the risk of dementia. Pharmacological interventions modulating β2-adrenoceptor activity might be a potential target in therapeutics for dementia.

## Key points

- A retrospective study was conducted to explore associations between β-adrenoceptor acting drugs and dementia in the older population.
- The use of β2-agonists was strongly associated with a decreased risk of dementia in a time-dependent manner.
- The use of non-selective β-antagonists significantly correlated with increased dementia risk.

## Introduction

Since the global population has continued to age, aging-associated cognitive disorders have already reached epidemic proportions. It is estimated that the age-standardized prevalence of dementia in people aged 60 years and older ranges from 4.7 to 8.7% in most countries (1). In China, the dementia prevalence was reported to be 6% aged 60 years or older, and the incidence was 9.87 cases per 1,000 person-years (2, 3). Increasing age is an unmodifiable risk factor for dementia, as aging is accompanied by numerous changes in the brain, including general atrophy and neuron loss in areas related to memory, an imbalance of amyloid-β production and degradation, activation of inflammation, alterations of neurotransmitters, such as noradrenaline, and so on (2). However, these age-related pathophysiologies are presented as potential targets for exploring treatment tactics for dementia. Locus coeruleus (LC), located in the brainstem and releasing noradrenaline throughout much of the brain, has been shown to participate in the modulation of a wide range of cognitive functions, such as episodic memory, working memory, executive function, learning and attention, vigilance, memory consolidation/retrieval, and cognitive reserve (4–6). Recent findings suggest that reduced integrity of LC is associated with declined cognition in late life (7, 8). As such, targeting noradrenergic activity would be a helpful strategy to slow down the progression of cognitive decline, and therefore numerous pharmacological interventions and clinical trials have been underway (9).

There are three types of adrenoceptors in the brain: α1, α2, and β transducing noradrenaline signals through downstream signaling pathways and influencing neuronal excitability and glial cell activity. In addition to the central nervous system, the subclasses of β-adrenoceptors are distributed broadly in the peripheral. For instance, β1-adrenoceptors are prevalent in the cardiomyocytes, β2-adrenoceptors are expressed mainly in the bronchial smooth muscle cells, whereas β3-adrenoceptors are found predominantly in adipose cells (10). Medications targeting the activity of β-adrenoceptors have currently been widely used in clinical practice. The β2-agonists are commonly involved in the treatment of respiratory diseases such as chronic obstructive pulmonary disease and asthma, reducing mortality worldwide, while β-antagonists, including non-selective β-antagonists and selective β1-antagonists, have usually been prescribed for hypertension, ischemic heart disease, chronic heart failure, tachyarrhythmia, migraine, and tremor (11–15). Most of the abovementioned diseases are highly prevalent in the older population.

Recently, studies considering the impact of β-adrenoceptor acting drugs on neurodegenerative diseases have attracted great interest. A group of epidemiological research investigated potential relationships between β-adrenoceptor acting drugs and Parkinson's disease (PD). They found that the chronic use of the β-antagonist was associated with an increased risk of PD, while the use of β2-agonists would decrease the risk (16). Strikingly, variations in the genes of β2-adrenoceptors have been reported to be involved in the pathogenesis of sporadic late-onset Alzheimer's disease (AD) (17). Since medications regarding β-adrenoceptors activity have commonly been applicated in the older population, we wondered whether β-adrenoceptor acting drugs would affect the risk of dementia in the older population. Thus, we conducted a retrospective case–control study to explore associations between β-adrenoceptor acting drugs and the risk of dementia in older adults, attempting to clarify the influence of β2-agonist and β-antagonist medications on cognitive decline.

## Methods

### Data sources

This study is based on data from the electronic medical record (EMR) database of the Department of Geriatrics, Ruijin Hospital, affiliated with the Shanghai Jiao Tong University School of Medicine. The EMR database contains comprehensive patient information, including demographics, International Classification of Diseases, Ninth Revision, and Tenth Revision (ICD-9 and ICD-10) diagnoses, procedures, prescription fills, and laboratory test results. We used both ICD-9 and ICD-10 codes to obtain patient diagnoses. The study period was from 1 January 2002 to 31 December 2019. The study was approved by the Medical Ethics Committee of Ruijin Hospital, affiliated with the Shanghai Jiao Tong University School of Medicine.

### Study design

#### Study population

We conducted a case–control study to investigate the association of β-adrenoceptor acting drugs with the risk of dementia aligns with the STROBE guidelines. The study consisted of all participants aged 60 years or older and alive on 1 January 2002 (entry date), with more than 1 year of follow-up. Individuals who used β-adrenoceptor acting drugs, as well as diagnosed with dementia before the entry date, were excluded. In addition, individuals with diagnoses of normal pressure hydrocephalus, intracranial tumor, massive cerebral infarction or hemorrhage, infarcts or hemorrhage in areas involved in cognition, and a history of neurosurgery were also excluded. We further excluded individuals with any previous record of a diagnosis of PD or PD-plus diseases, including multiple system atrophy, progressive supranuclear palsy, frontotemporal lobar degeneration, and Lewy body dementia. All participants were followed until reaching the study outcome (development of dementia), death, loss to follow-up, or end of follow-up on 31 December 2019, whichever came first.

#### Exposure data

The use of β2-agonists and β-antagonists was determined based on EMR database records. The β2-agonists and β-antagonists involved in this study were the drugs that were commonly applied in mainland China during the observational period. In our clinical practice, β2-agonists include short-acting β2-agonists (SABAs) salbutamol and terbutaline, long-acting β2-agonists (LABAs) salmeterol and formoterol, and ultra-long-acting β2-agonists (ultra-LABAs) vilanterol. The category of β-antagonist drugs contains non-selective β-antagonists including propranolol, carvedilol, sotalol, and arotinolol, and selective β1-antagonists including metoprolol, bisoprolol, and atenolol. Participants who used β2-agonists or non-selective β-antagonists for more than 6 months and with a prescribed duration recorded more than 1 year before the end point were grouped as cases. Participants who never used β2-agonists or non-selective β-antagonists, or used β2-agonists or non-selective β-antagonists <6 months, or with prescribed duration recorded <1 year before the end point were grouped as controls.

#### Outcomes

Participants identified as having dementia during the observational period were regarded as reaching study outcomes. Identification of dementia was determined by records of diagnostic ICD codes or prescriptions of anti-dementia drugs. In parallel, the onset of dementia was defined as the first medical encounter time for a diagnosis of dementia in our database.

#### Covariates

Demographic information included age, sex, schooling years, and follow-up time was seen as baseline variables. Potential confounders, including alcohol consumption, smoking history, stroke (especially referred to as focal infarcts or hemorrhage in areas not involved in cognition), and diabetes, known to be associated with dementia risks, were collected. Comorbidities indicative of medications of β2-agonists (respiratory diseases involving chronic obstructive pulmonary disease and asthma) and β-antagonists (essential tremor, hypertension, heart diseases involving coronary heart disease, atrial fibrillation, and chronic heart failure) were also investigated.

### Statistical analysis

A propensity score matching strategy was employed to select matched control participants for β2-agonist and β-antagonist users, respectively. Regarding β2-agonist users, control subjects were matched by baseline age, sex, follow-up time, schooling years, alcohol consumption, smoking history, heart disease, hypertension, and diabetes. Similarly, for β-antagonist users, matched control groups were set considering covariates including age, sex, follow-up time, schooling years, alcohol consumption, smoking history, respiratory diseases, and diabetes. Each β2-agonist or non-selective β-antagonist user was matched to five control subjects with the nearest neighbor matching within a caliper width of 0.20, while the matching of selective β1-antagonist users was set with a ratio of 1:1. Standardized mean difference (SMD) was used to judge the differences of covariate after performing propensity-score matching. SMD <0.10 indicated few or trivial differences between groups. Propensity-score matching was performed using the R MatchIt package (version 4.1.1).

All continuous data were presented as mean ± standard deviation (SD) and compared using a *t*-test. Categorical data were presented as N (%) and compared using the chi-square test. Logistic regression was conducted to estimate the odds ratio (OR) of dementia and its 95% confidence interval (95% CI) with the use of β2-agonists by adjusting for stroke, respiratory diseases, and the use of β-antagonists, while with the use of β-antagonists by controlling confounders of essential tremors, heart diseases, hypertension and the use of β2-agonists. For selective β1-antagonist usage, covariates including stroke, heart diseases, hypertension, and the use of β2-agonists were adjusted during logistic regression analysis. A *P* < 0.05 (two-tailed) was considered statistically significant. We further performed a sensitivity analysis to explore a possible time–response relationship between dementia and the use of β2-agonists or β-antagonists. The additional sensitivity analyses comprised the risk of dementia associated with having never used, cumulatively used <24 months, and cumulatively used more than 24 months medications of β2-agonists or β-antagonists. The test for trend was based on variables containing a median value for each quintile. All statistical analyses in this study were performed using R (version 4.1.1) with R Studio (version 1.1.442).

## Result

**Figure 1:**
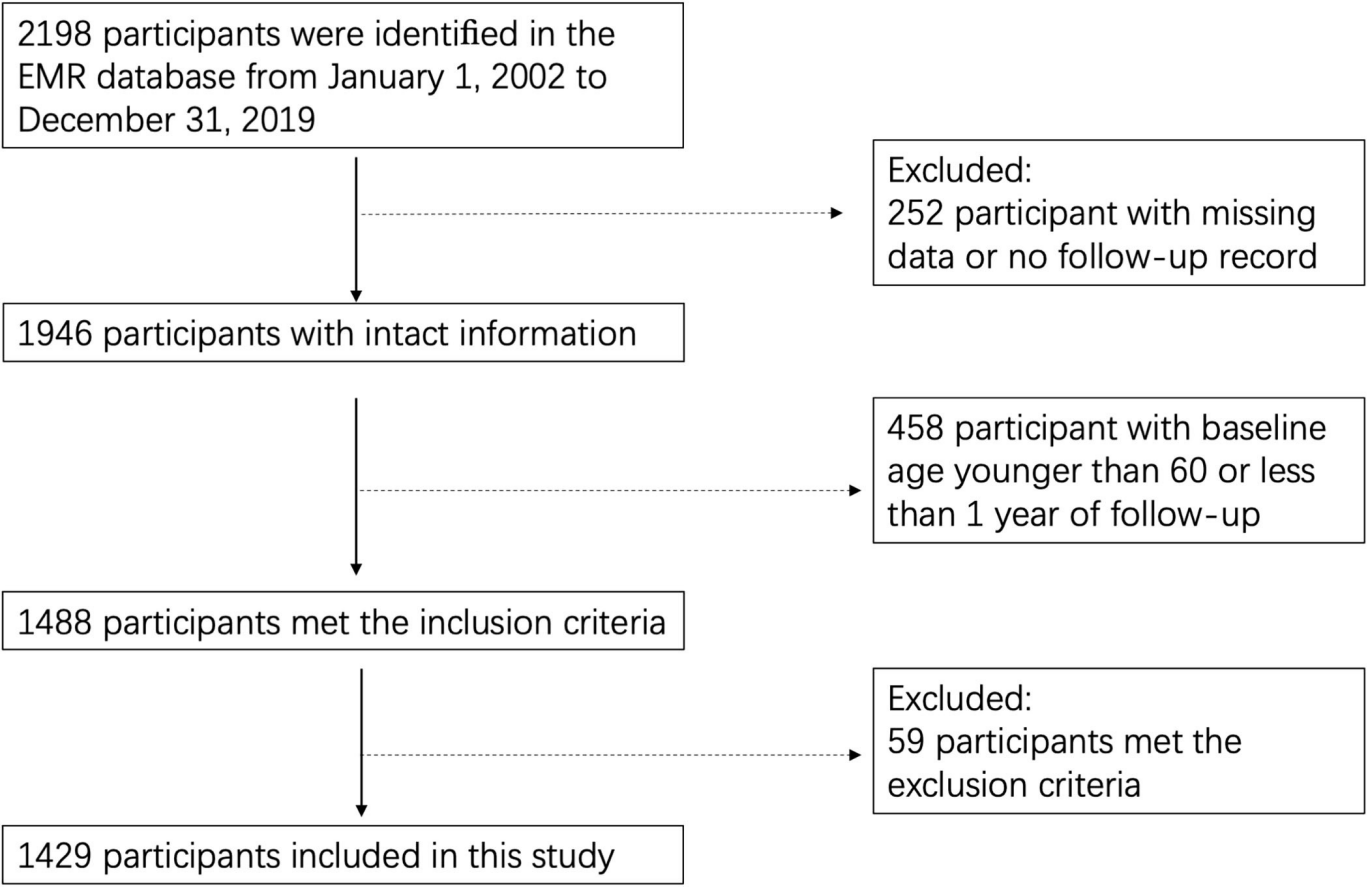
Flowchart displaying a selection of the study population.

A total of 2,198 adults were identified in the EMR database from 1 January 2002 to 31 December 2019. Among them, 252 individuals were removed for missing records, while 458 were removed since their baseline age was younger than 60 or the duration of follow-up was <1 year. We further excluded 59 individuals for their recorded dementia or usage of β2-agonists or β-antagonists before the entry date, as well as for their history of normal pressure hydrocephalus, intracranial tumor, massive cerebral infarction or hemorrhage, infarcts or hemorrhage in areas involved in cognition, undergoing neurosurgery or diagnoses of other neurodegenerative diseases, such as PD. The remaining 1,429 individuals met our eligibility criteria and were included in this study (Figure 1). For all participants, the mean baseline age was 73.2 ± 7.9 years with a mean follow-up time of 9.2 ± 4.3 years. Notably, 84.0% of the study population were men. The average schooling year of participants included was 15 ± 2 years, and about 87% have a college degree or above. During follow-up, 215 (14.8%) participants were diagnosed with dementia, including 110 with AD, 51 with vascular dementia, and 54 with mixed-type dementia. Prescription records showed that 100 (7.0%) participants used β2-agonists, while 112 (7.7%) used β-antagonist medications.

For β2-agonist users, propensity-score matching using a greedy nearest-neighbor matching within a specified caliper distance of 0.2 was performed to find matched controls. As shown in Table 1, 446 control subjects were matched to 98 β2-agonist users with a total standardized mean difference (SMD) of 0.010. Variables, including age, sex, follow-up time, schooling years, alcohol consumption, smoking history, heart diseases, hypertension, and diabetes, were balanced between the two groups. No significant differences were found in stroke and consumption of β-antagonists between β2-agonist users and non-users. As the main indications for β2-agonist drugs, respiratory diseases occurred obviously more commonly in the group of β2-agonist users than in non-users.

**Table 1:** Characteristics of β2-agonist users and their matched controls.

	**β2-agonist users** **(*N* = 98)**	**β2-agonist non-users (*N* = 446)**	***P*-value**	**SMD**
Age (SD)	73.43 (6.93)	73.05 (7.30)	0.643	0.033
Male (%)	89 (90.8)	395 (88.6)	0.519	0.041
Schooling years			0.921	0.008
Schooling year ≤ 12 years (%)	16 (16.3)	71 (15.9)		
Schooling year >12 years (%)	82 (83.7)	375 (84.1)		
Follow-up time (SD)	11.28 (3.52)	11.15 (3.87)	0.777	0.036
Alcohol (%)	6 (6.1)	31 (7.0)	0.768	0.026
Smoking (%)	45 (45.9)	183 (41.0)	0.375	0.001
Heart disease (%)	41 (41.8)	184 (41.3)	0.916	0.001
Respiratory disease (%)	83 (84.7)	136 (30.5)	<0.001	/
Hypertension (%)	76 (77.6)	362 (81.2)	0.413	0.094
Diabetes (%)	31 (31.6)	149 (33.4)	0.735	0.020
stroke (%)	8 (8.2)	60 (13.5)	0.152	/
Dementia (%)	9 (9.2)	67 (15.0)	0.131	/
β-antagonist users (%)	6 (6.1)	34 (7.6)	0.606	/

SD, standard deviation; SMD, standardized mean difference.

The association between the use of β2-agonists and the risk for dementia was analyzed by logistic regression controlling for respiratory diseases, stroke, and the use of β-antagonists. Strikingly, the consumption of β2-agonists was shown to be associated with a decreased risk of dementia (OR = 0.324, 95% CI: 0.149–0.707, *P* = 0.005). Further sensitivity analysis demonstrated a time–response manner. Reduced risks were seen for both short-term use (≤ 24 months) and long-term use (>24 months) (OR = 0.380 and 0.211, respectively), indicating dementia risk decreased in a statistically significant time-dependent pattern with increased medication of β2-agonists (*P*_*trend*_ = 0.014 ) (Table 2). As most of the sample included in our study were male participants, we further performed a stratified analysis by sex. In male participants, the use of β2-agonists was associated with a decreased risk of dementia (OR = 0.347, 95% CI: 0.157–0.767, *P* = 0.009). For the female participants, the analysis was unavailable because of the limited sample after propensity-score matching.

**Table 2:** Association between β2-agonist users and risk of dementia.

	**OR (95% CI)^a^**	***P*-value**
**Exposure group**		
β2-agonist non-user	1	
β2-agonist user	0.324 (0.149–0.707)	0.005
**Cumulative use, months**		
Never	1	
≤ 24	0.380 (0.150–0.960)	0.041
>24	0.211 (0.061–0.723)	0.013
*P*-value for trend	0.954 (0.919–0.990)	0.014

a Adjusted for stroke, respiratory disease, and use of β2-agonists; CI, confidence interval; OR, odds ratio.

As mentioned earlier, 112 β-antagonist users were matched with 553 control subjects using the propensity score matching technique with a total SMD of 0.006. Variables, including age, sex, follow-up time, schooling years, alcohol consumption, smoking history, respiratory diseases, and diabetes, were balanced between the two groups. There were no significant differences in stroke and consumption of β2-agonists in β-antagonist users compared with β-antagonist non-users. Heart diseases, hypertension, and essential tremor, which are indicative of β-antagonist medications, accounted for significantly higher proportions in β-antagonist users as expected (Table 3). In the analysis of logistic regression, the use of β-antagonists was associated with an increased risk of dementia (OR = 1.961, 95% CI 1.144–3.359, *P* = 0.014) after controlling for stroke, heart diseases, hypertension, essential tremor, and the use of β2-agonists. However, a time-dependent effect was not found between the medication of β-antagonists and dementia risk (*P*_*trend*_ = 0.220) (Table 4). We also performed a stratified analysis by sex for β-antagonist users. We found that the medication of β-antagonists was associated with an increased risk of dementia in trend among male and female participants separately (male: OR = 1.762, 95% CI: 0.951–3.264, *P* = 0.072; female: OR = 3.136, 95% CI: 0.956–10.284, *P* = 0.059).

**Table 3:** Characteristics of β-antagonist users and their matched controls.

	**β2-antagonist users (*N* = 112)**	**β2-antagonist non-users (*N* = 553)**	***P*-value**	**SMD**
Age (SD)	74.01 (7.05)	73.61 (7.44)	0.700	0.055
Male (%)	94 (83.9)	458 (82.8)	0.776	0.034
Schooling years			0.924	0.006
Schooling year ≤ 12 years (%)	17 (15.2)	82 (14.8)		
Schooling year >12 years (%)	95 (84.8)	471 (85.2)		
Follow-up time (SD)	11.13 (3.75)	11.10 (3.83)	0.938	0.005
Alcohol (%)	6 (5.4)	28 (5.1)	0.898	0.010
Smoking (%)	29 (16.4)	83 (17.0)	0.849	0.019
Heart disease (%)	78 (69.6)	235 (42.5)	<0.001	/
Respiratory disease (%)	39 (34.8)	184 (33.3)	0.752	0.027
Hypertension (%)	105 (93.8)	455 (82.3)	0.002	/
Diabetes (%)	58 (51.8)	285 (51.5)	0.962	0.003
Essential tremor (%)	13 (11.6)	10 (1.8)	<0.001	/
Stroke (%)	16 (14.3)	62 (11.2)	0.357	/
Dementia (%)	27 (24.1)	71 (12.8)	0.002	/
β2-agonist users (%)	6 (5.4)	45 (8.1)	0.313	/

SD, standard deviation; SMD, standardized mean difference.

**Table 4:** Association between β-antagonist users and risk of dementia.

	**OR (95% CI)^a^**	***P*-value**
**Exposure group**		
β-antagonist non-user	1	
β-antagonist user	1.961 (1.144–3.359)	0.014
**Cumulative use, months**		
Never	1	
≤ 24	2.215 (1.110–4.067)	0.023
>24	1.597 (0.747–3.415)	0.227
*P*-value for trend	1.007 (0.996–1.017)	0.220

a Adjusted for stroke, essential tremor, heart disease, hypertension, and use of β2-agonists CI, confidence interval; OR, odds ratio.

Similar analyses were also conducted for the group of selective β1-antagonist users. Matched controls were selected by the same covariates resembling non-selective β-antagonist users but with a ratio of 1:1 since 442 participants only used β1-antagonists. Interestingly, a null association was found between dementia risk and the use of selective β1-antagonists after adjusting for stroke, heart diseases, hypertension, and the use of β2-agonists (OR = 1.114, *P* = 0.625).

## Discussion

This study showed that the use of β2-agonists was associated with a decreased risk of dementia in a time–response manner, whereas the use of non-selective β-antagonists increased dementia risk. In addition, selective β1-antagonists seem to lack associations with the risk of dementia, indicating the effects of increasing dementia risk may be related to the β2-antagonists part in non-selective β-antagonists.

At present, the propensity-score technique has gained much popularity for its ability to reduce systematic bias for observational studies by balancing the distribution of prognostically important covariates between comparator groups, and thus has been used in numerous cardiovascular research (18). For our study, in order to control the potential confounding effect of the drug itself, as well as covariates including age, sex, schooling years, smoking history, alcohol consumption, and comorbidities, such as diabetic state, hypertension, heart, and respiratory diseases, which are known to be epidemiological risk factors for dementia (2, 19) and may mislead the results, we used propensity-score analysis to match β2-agonists or β-antagonists users with controls from non-users, respectively.

Inhaled β2-agonists have commonly been prescribed in the treatment of respiratory diseases such as chronic obstructive pulmonary disease and often in concomitant with prescriptions of anticholinergics. Despite the anticholinergic drugs might exert a negative impact on cognition, our results demonstrated that the use of β2-agonists would decrease the risk of dementia (20, 21). Further analysis revealed a significant correlation of incremental duration of β2-agonists usage with corresponding reductions of ORs, supporting the notion that β2-agonist drugs might have cumulative preventive effects in the development of dementia.

The protective roles of β2-adrenoceptors activation have been confirmed by a series of studies recently. β2-agonist drugs such as formoterol were shown to be capable of reducing oxidative stress, attenuating apoptosis, promoting dendritic complexity, and improving cognition in AD-related mouse models (22, 23). Activation of β2-adrenoceptors may decrease AD-related pathologic proteins and prevent their impairment. A recent study using circular dichroism spectroscopy identified salbutamol as an inhibitor of tau protein aggregation *in vitro* (24). Chai et al. reported that the activation of β2-adrenoceptors could decrease cerebral amyloid plaque burden through increased α-secretase activity (25). The finding from a study conducted by Li et al. indicated that the activation of β2-adrenoceptors and downstream signaling pathways could facilitate the long-term potentiation of hippocampal neurons. Moreover, chronically fed wild-type mice with drugs acting on β2-adrenoceptors could protect the hippocampus from impairment by amyloid-beta oligomers (26). The activation of β2-adrenoceptors could also be implicated in the modulation of neuroinflammation. Direct stimulation of glial β2-adrenoceptors by agonists would suppress the expression of proinflammatory genes, induce the expression of anti-inflammatory mediators, and enhance the production of neurotrophic factors (23, 27). Therefore, the inhibition of β2-adrenoceptors could exert a detrimental effect on AD-like pathology and exacerbate cognitive deficits (28).

In our study, with adjustment for hypertension, as well as heart diseases and essential tremor, on which non-selective β-antagonists are usually prescribed as above mentioned, we found that an increased risk of dementia was associated with the use of non-selective β-antagonists. Although no further time-dependent association was found, the following results of a null association between selective β1-antagonists use and dementia indicated the increased risk by the use of non-selective β-antagonists obtained in the present study might not be due to confounding factors. Partially supporting our results, a previous study exploring the influence of β-blockers, including propranolol, carvedilol, and metoprolol, on cognitive function revealed a trend for worse delayed memory retrieval in cognitively impaired patients (29). In their discussion, they agreed that specified analysis for subgroups of β-blockers and a balanced distribution of vascular risk factors are required for future investigation. In addition, the discrepant ability of β-antagonist drugs to cross the blood–brain barrier (BBB) should be addressed. For non-selective β-antagonists, propranolol and carvedilol are classified with high BBB permeability, arotinolol with moderate BBB permeability, and sotalol with low BBB permeability (30). For selective β1-antagonists, metoprolol is classified with moderate BBB permeability, bisoprolol, and atenolol with low BBB permeability, while none with high BBB permeability (30). Thus, the inconsistent findings of non-selective β-antagonists and selective β1-antagonists may be related to the diverse BBB permeability, as β-antagonists with high or moderate BBB permeability may exert central effects. Studies with larger samples for exploring associations between different β-antagonist drug categories and the risk of dementia are needed in future. However, several previous studies and meta-analyses for associations between hypertensive medication and cognitive impairment in the older population found β-blockers were associated with a lower rate of cognitive decline (31, 32). However, other antihypertensive medications analyzed in these studies also exhibited similar associations, reflecting an overall benefit of positive controlling of blood pressure and its comorbidities for cognition, as indicated by the SPRINT-MIND trial that intensive blood pressure control was associated with a reduction in mild cognitive impairment and a statistically non-significant reduction in the risk of probable dementia as well as a smaller increase in cerebral white matter lesion volume (33, 34). In addition, we must address the bias of essential tremor, hypertension, and heart disease between the groups because β2-antagonists may be prescribed for these diseases, which are hard to avoid and could confound the results. Thus, the finding of the association between non-selective β-antagonists and dementia should be interpreted prudently.

To our knowledge, this study is the first longitudinal study to explore the association between β2-adrenoreceptor medications and the risk of dementia in the older population. In the current observational research, the mean follow-up time was 9.2 ± 4.3 years, which was even more than 10 years in each analyzed group after matching, thus allowing us to investigate the long-term effects of β2-adrenoreceptor medications on dementia risk. In addition, all participants were 60 years old, with the mean age being 73.2 ± 7.9 years, facilitating a specified illustration of the possible influence of β-adrenoreceptor acting drugs in older adults. More importantly, the propensity score matching technique performed in this longitudinal study for patient cohort selection has provided assurance for better control of confounders than traditional covariate adjustment methods and, therefore, may add more strength for validating our results. Still, the study subjects consist predominantly of male participants, and a relatively small sample size from one department may limit its intended interpretation for a general population. However, we controlled for biological sex by propensity-score matching, and the proportion of male or female participants was balanced between case and control groups. In addition, we also performed stratified analysis by sex and still found the use of β2-agonists was associated with a decreased risk of dementia in male participants, and β-antagonist drug usage was associated with an increased risk of dementia in male and female participants separately in trend. The similar tendency of β-antagonist drugs with dementia in male and female participants suggested that β-antagonist medication was associated with increased dementia risk regardless of biological sex and may be related to the limited sample of each sex, especially female participants. Thus, the significant association between β2-adrenoreceptor medications and the risk of dementia was with limited bias. Because of the predominance of male participants in our study and the higher risk of AD among female participants, the association may be underestimated. Further studies, including more female participants or an equivalent ratio of sexes, were needed to investigate the effect of β2-adrenoreceptor medications on dementia in different sexes. In addition, diagnosis of dementia based on ICD codes may lead to the loss of substantial numbers of individuals in the early prodromal stage of dementia. As such, longitudinal studies with expanded sample size and balanced sex ratio from multicenter, in combination with neuropsychological assessment and neuroimaging technique targeting alterations in regions including LC and hippocampus to describe the impact of β-adrenoreceptor acting drugs, especially β2-agonist medications, on cognitive function are in urgent need.

## Conclusion

In summary, results from this case–control study presented significant associations between β-adrenoreceptor acting drugs and the risk of dementia. Our data provided evidence that β2-agonist drugs are associated with lower dementia risk and could be a potential target for developing neuroprotective medications in the therapeutics for dementia. We also recommend that it would be prudent to evaluate the risk–benefit ratio before prescribing medications of β-antagonists. Moreover, cognitive assessment before the initiation of these agents and regularly repeated during the follow-up time might be required in the older population.

## Data availability statement

The raw data supporting the conclusions of this article will be made available by the authors, without undue reservation.

## Ethics statement

The studies involving human participants were reviewed and approved by Ethics Committees of Shanghai Ruijin. The patients/participants provided their written informed consent to participate in this study.

## Author contributions

SC performed the statistical analysis and wrote the paper. FF collected the data and revised the paper. PC assisted with writing the article. QJ and SX supervised the data collection. ZX and JH formulated the research question and designed the study. FL designed the study and revised the article. All authors contributed to the article and approved the submitted version.
